# MLVA genotyping of Chinese human *Brucella melitensis *biovar 1, 2 and 3 isolates

**DOI:** 10.1186/1471-2180-11-256

**Published:** 2011-11-22

**Authors:** Hai Jiang, Mengguang Fan, Jingdiao Chen, Jingchuan Mi, Ruiping Yu, Hongyan Zhao, Dongri Piao, Changwen Ke, Xiaoling Deng, Guozhong Tian, Buyun Cui

**Affiliations:** 1 State Key Laboratory for Infectious Disease Prevention and Control, National Institute for Communicable Disease Control and Prevention, Chinese Center for Disease Control and Prevention, 155 Changbai Road, Changping, Beijing 102206, PR China; 2 Department of Brucellosis, Inner Mongolia Center of Endemic Disease Control and Research, 50 Erdos Street, Huhhot 010031, PR China; 3 Department of Bacterial Pathogens, Institute for Pathogenic Microorganisms, Guangdong Center for Disease Control and Prevention, 176 Newport Road West, Guangzhou 510300, PR China

## Abstract

**Background:**

Since 1950, *Brucella melitensis *has been the predominant strain associated with human brucellosis in China. In this study we investigated the genotypic characteristics of *B. melitensis *isolates from China using a multiple-locus variable-number tandem-repeat analysis (MLVA) and evaluated the utility of MLVA with regards to epidemiological trace-back investigation.

**Results:**

A total of 105 *B. melitensis *strains isolated from throughout China were divided into 69 MLVA types using MLVA-16. Nei's genetic diversity indices for the various loci ranged between 0.00 - 0.84. 12 out 16 loci were the low diversity with values < 0.2 and the most discriminatory markers were bruce16 and bruce30 with a diversity index of > 0.75 and containing 8 and 7 alleles, respectively. Many isolates were single-locus or double-locus variants of closely related *B. melitensis *isolates from different regions, including the north and south of China. Using panel 1, the majority of strains (84/105) were genotype 42 clustering to the 'East Mediterranean' *B. melitensis *group. Chinese *B. melitensis *are classified in limited number of closely related genotypes showing variation mainly at the panel 2B loci.

**Conclusion:**

The MLVA-16 assay can be useful to reveal the predominant genotypes and strain relatedness in endemic or non-endemic regions of brucellosis. However it is not suitable for biovar differentiation of *B. melitensis*. Genotype 42 is widely distributed throughout China during a long time. Bruce 16 and bruce 30 in panel 2B markers are most useful for typing Chinese isolates.

## Background

Brucellosis, recognized as a common zoonotic disease globally, is caused by bacteria of the genus *Brucella*. *B. melitensis*, *B. abortus*, and *B. suis *remain the principal causes of human brucellosis worldwide and are major public health problems, primarily in Africa, the Middle East and Southeast Asia [1]. Brucellosis is prevalent in China, especially in the northern China, where people are economically dependent on ruminant livestock. Approximately 30,000 human cases are reported annually over the past 5 years [2]. In China, *B. melitensis *was the predominant strain associated with human brucellosis outbreaks, according to annual report on surveillance of selected infectious disease and vector. Species identification and subtyping of *Brucella *isolates is very important for epidemiologic surveillance and investigation of outbreaks in *Brucella*-endemic regions [3,4].

Recent studies have confirmed that multiple-locus variable-number tandem-repeat analysis (MLVA) is a useful tool for identifying and genotyping *Brucella *strains and the resultant data can be used for epidemiological trace-back investigations [3,5-8]. In efforts to better improve surveillance and evaluate the power of epidemiological trace-back in China, the MLVA-16 scheme was used to type a collection of 105 *B. melitensis *isolates from 18 different regions throughout China.

(This study was presented in part at the 5th Brucellosis International Research Conference of the American Society for Microbiology, Buenos Aires, Argentina, 2011.)

## Results

### Typing and clustering of *B. melitensis *isolates by MLVA-16

**Figure 1:**
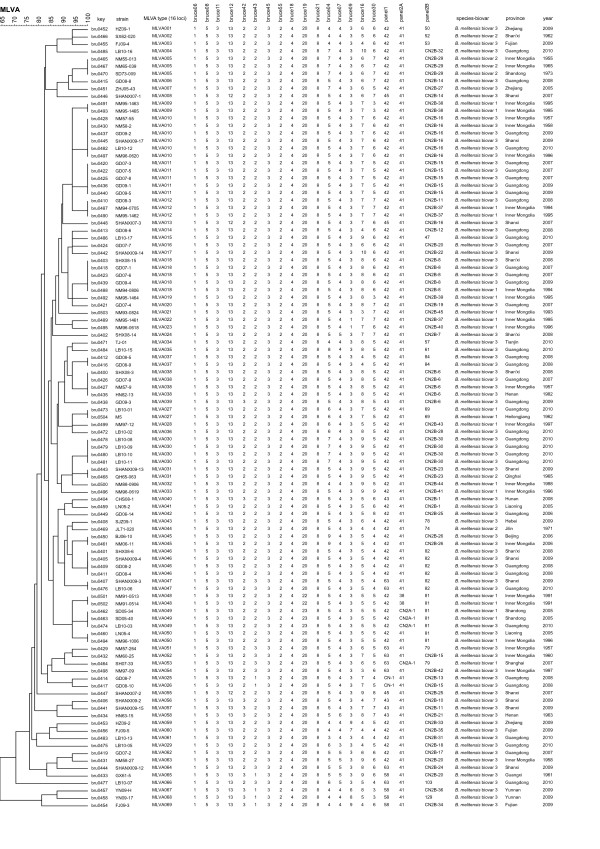
**Dendrogram based on the MLVA-16 genotyping assay showing relationships of the 105 *B. melitensis *isolates**. MLVA type: panel 1 and panel 2 genotypes in this article; key: serial number for the isolate in the Brucella2010 MLVA database http://mlva-u-psud.fr/; strain: strain name in the laboratory in which the DNA extraction was done; province/year: province and year of isolation; panel1, panel 2A, panel 2B: genotypes corresponding to each isolates in the database for each set of loci; The actual biotyping result is indicated in the species-biovar column.

Using the complete MLVA-16 assay (including panel 1, 2A and 2B loci), the 105 *B. melitensis *isolates were clustered in 69 different genotypes with 17 clusters and 52 singleton genotypes (Figure 1). The corresponding diversity index for panels 1, 2A, and 2B were 0.37, 0.11, and 0.98 respectively. The overall discriminatory index of MLVA-16 in this population was 0.99. Using panel 1, the present population clustered into five known genotypes and a new genotype. The five known genotypes were included in the previously named the 'East Mediterranean' group with genotypes 42 (83 strains), 43(5 strains), 45(3 strains), 58(4 strains) and 63(8 strains). All were included in the previously recognized 'East Mediterranean' group. Two strains from Guangdong, isolated in 2008, had the genotype (1-5-3-13-2-1-3-2), labeled as CN-1. The two strains were a single-locus variant (SLV) to genotype 42(1-5-3-13-2-2-3-2). To date the genotype associated with CN-1 has not been reported from any other country.

Greater diversity among the Chinese *B. melitensis *isolates was apparent when the eight additional markers encompassing panel 2A and 2B were included. The number of strains populating a cluster ranged from two (eight clusters) to six. Clusters comprised of two strains were as follows: MLVA type009 (1-5-3-12-2-2-3-2-4-20-8-7-4-3-6-7), MLVA type027 (1-5-3-12-2-2-3-2-4-20-8-6-4-3-7-5), MLVA type031 (1-5-3-12-2-2-3-2-4-20-8-5-4-3-9-5), MLVA type037 (1-5-3-12-2-2-3-2-4-20-8-5-4-3-4-5), MLVA type045 (1-5-3-12-2-2-3-2-4-20-8-9-4-3-5-4), MLVA type047 (1-5-3-12-2-2-3-2-4-20-8-5-4-3-5-4), MLVA type048 (1-5-3-12-2-2-3-2-4-22-8-5-4-3-5-5) and MLVA type050 (1-5-3-12-2-2-3-2-4-20-8-5-4-3-5-5). Clusters comprised of three strains were as follows: MLVA type005 (1-5-3-12-2-2-3-2-4-20-8-7-4-3-6-7), MLVA type012 (1-5-3-12-2-2-3-2-4-20-8-5-4-3-7-7) and MLVA type049 (1-5-3-12-2-2-3-2-4-23-8-5-4-3-5-5). Clusters comprised of four strains were as follows: MLVA type030 (1-5-3-12-2-2-3-2-4-20-8-7-4-3-9-5), MLVA type038 (1-5-3-12-2-2-3-2-4-20-8-5-4-3-8-5) and MLVA type046 (1-5-3-12-2-2-3-2-4-20-8-5-4-3-5-4). Clusters comprised of five strains were as follows: MLVA type011 (1-5-3-12-2-2-3-2-4-20-8-5-4-3-7-5) and MLVA type018(1-5-3-12-2-2-3-2-4-20-8-5-4-3-8-6). Cluster comprised of six strains was MLVA type010 (1-5-3-12-2-2-3-2-4-20-8-5-4-3-7-6). Based upon the year of isolation, it is evident that many of the genotypes identified using MLVA-16 appear to have persisted for a long time in China and may be associated with spread of the strains from northern to southern China; more data will need to be collected to re-enforce these observations. The most discriminatory markers were bruce16 and bruce30 of panel 2B, with a diversity index of > 0.75 harboring 8 and 7 alleles, respectively. The most homogeneous markers, in contrast, were bruce06, bruce08, bruce11, bruce18, bruce21, bruce45 and bruce55 of panel 1 and panel 2A. The main characteristics of the 16 loci in the 105 *B. melitensis *strains are shown in Table 1.

**Table 1:** Main characteristics of 16 VNTR loci in 105 *B. melitensis *isolates

Locus	Repeat size(bp)	No. of alleles	No. of repeats	Nei's DI and 95%CI
bruce06	134	1	1	0.00
bruce08	18	1	5	0.00
bruce11	63	1	3	0.00
bruce12	15	2	12-13	0.07(0.00-0.14)
bruce42	125	2	2-3	0.17(0.08-0.26)
bruce43	12	3	1-3	0.25(0.15-0.36)
bruce45	18	1	3	0.00
bruce55	40	1	2	0.00
bruce18	8	1	4	0.00
bruce19	6	3	20,22-23	0.13(0.04-0.21)
bruce21	8	1	8	0.00
bruce04	8	7	3-9	0.47(0.36-0.58)
bruce07	8	4	3-6	0.16(0.07-0.25)
bruce09	8	6	1,3,6-9	0.13(0.04-0.23)
bruce16	8	8	3-10	0.83(0.81-0.85)
bruce30	8	7	3-9	0.75(0.71-0.79)

### Analysis of the isolates from Inner Mongolia and Guangdong

Using the complete MLVA-16 assay, the 26 *B. melitensis *isolates from Inner Mongolia and 39 isolates from Guangdong were clustered in 20 and 27 different genotypes, respectively. The bruce16 loci had 6 and 7 alleles and the bruce30 loci had 6 and 5 alleles in these two population. 7 genotypes of 11 isolates from Inner Mongolia and 9 genotypes of 16 isolates from Guangdong isolates were single-locus or double locus variants of the main subgroups at 90% similarity. Only two of the clusters consisted of epidemiologically related strains exclusively from the same province and the same time. Bru0501 and bru0502 isolates came from a married couple who contacted with sheep fetuses and placenta in May, 1991 from Bai country, Xilinguole, Inner Mongolia. Bru0478, br0479, bru0480 and bru0481 isolates were collected over six weeks from late April to early June, 2010, in Pingsha county, Zhuhai city, Guangdong province. These four patients had not identified at-risk occupational history and experienced fever, debility and joint pains.

### Trace-back of a laboratory-acquired *Brucella *infection

We report a case of brucellosis affecting a hospital microbiology laboratory technician in Beijing, a non-endemic area of China. To better elucidate the origin of such infection, *Brucella *strains from both the patient and the laboratory technician were characterized by MLVA-16. The strain BJ06-10 showed the same MLVA type with strain NM06-11 isolated from a patient with acute brucellosis who engaged in fur-making in Inner Mongolia.

### Identification of the *B. melitensis *vaccine strain M5

LB10-01, a *B. melitensis *biovar 1 strain isolated from Guangdong in 2010 was indistinguishable from the vaccine strain M5 according to the MLVA cluster analysis (MLVA027: 1-5-3-13-2-2-3-2-4-20-8-6-4-3-7-5). This is unexpected since the vaccine strain M5 was not used in Guangdong. Detection of a strain with phenotypic and genotypic properties indistinguishable from the vaccine strain M5 raises the concern of the origin of the wild type strain.

## Discussion

Brucellosis surveillance was started in 1980 in some parts of China. In 2008, 21 surveillance points for animal and human brucellosis were established in the 19 provinces of Heilongjiang, Jilin, Hebei, Henan, Inner Mongolia, Shandong, Guangdong, Guangxi, Sichuan, Tibet, Gansu, Ningxia, Xinjiang, Shanxi, Shan'xi, Zhejiang, Liaoning, Ningxia and Yunnan. Since the established of these surveillance points more than 30 years ago, a huge panel of animals and humans strains have been surveyed. It is significant that the national epidemiological characteristics can be analyzed. It suggests that *B. melitensis *isolates from different locations and years would reflect the epidemic features of human brucellosis.

Sheep infected with *Brucella *are one of the main sources for human and animal brucellosis in China [9]. Over the last 20 years, the geographic distribution of brucellosis in China had been changing from pasturing areas to regions of with reduced agricultural interests (or alternatively more industrial concentrations); in these areas the infection rates, reported incidence, and number of outbreaks of brucellosis have increased markedly based on the National Notifiable Disease Surveillance System data. During this period, the cases have mostly been reported from Inner Mongolia, Shanxi, Hebei, Shandong, Henan, Liaoning, Jilin, Heilongjiang, and Shan'xi provinces. It is worth noting that brucellosis is endemic in Guangdong province, one of the wealthiest and industrial provinces in China. This is because of the movement of infected animal to Guangdong, resulting in the change of the geographic distribution of brucellosis.

In the different epidemic regions of China, the predominant strains have been shown to be *B. melitensis *biovar 1, 2, or 3 [9]. Since 2005, most human cases in China have been caused by *B. melitensis *biovar 3 [10]. Classical typing systems are unable to subdivide *Brucella *isolates below the biovar level. Molecular typing methods such as MLVA have been utilized to distinguish between strains of the same biovar in both animal and human isolates [3,5,6,11-13]. In an effort to assess the value of MLVA as a subtyping tool for *Brucella *strains, genotypic characteristics of 105 *B. melitensis *isolates were investigated. Cluster analysis of these China strains, based on the eight variable-nucleotide tandem repeat loci included in the MLVA-16 panel 1 grouped them all into the *B. melitensis *'East Mediterranean group' [3] and unique from circulating strains in Northern Africa, Southern Europe ('West Mediterranean group' and 'American group'). For instance, an (panel 1 genotype 42 and 43) clustered separately from most of the other 'West Mediterranean group' (panel 1 genotype 49 and 51) and 'American group'(panel 1 genotype 47). Previous studies have shown that Near Eastern countries frequently report human cases associated with genotypes 42 and 43 [3,14]. Genotype 42, as we have shown, is widely distributed throughout China, and has previously been reported to be predominant in Turkey, Portugal and Spain [13]. In Spain, human *B. melitensis *strains clustered into genotypes 42 (Eastern Mediterranean group, 55%), 48 and 53 (Americas group, ~11%) and 51 (Western Mediterranean group, ~8%). Chinese *B. melitensis *are classified in limited number of closely related genotypes showing variation mainly at the panel 2B loci.

In China, the Inner Mongolia Autonomous Region is the most severe endemic focus of brucellosis, with an annual incidence of the disease varying from 40 to 70/100,000 during 2005-2010 [2]. Inner Mongolia is in close proximity to Heilongjiang, Jilin, Hebei and Shanxi provinces; these provinces are located in the north and east of China, where stocking raising is the most important aspect of the economy. In these regions, *B. melitensis *genotype 42 strains were predominant, but genotype 42 strains were also common in provinces reporting sporadic cases such as Liaoning, Shandong, Zhejiang, Fujian and Tianjin. These isolates were only single-locus or double-locus variants of *B. melitensis *from the endemic regions. Of particular note is the apparently stability of genotype 42 in China; genotype 42 strains were isolated from Inner Mongolia in1957 as well as 53 years later.

Guangdong province, which is now considered to be an endemic region for brucellosis, is located in the southern coastal region of China, where the incidence of human brucellosis has increased gradually since 2000. The prevailing panel 1 type is genotype 42 as well. The genotypes for most of the *B. melitensis *isolates in this series and their close relatedness by MLVA (single-locus variants and in some cases double-locus variants) compared to the relatedness of *B. melitensis *isolates in other countries reflects microevolution within the regionally important strain due to a few mutational events [3]. We also observed that strains from the north and east of China (eg., Inner Mongolia and Shanxi) had the same MLVA-16 genotype (010) as those from the south of China (eg., Guangdong). This data indicates that the emergence of brucellosis in the south of China is likely to have its origins from the importation of animals from elsewhere in China. The clustering of epidemiologically-related isolates identified in the current and previous studies support the use of MLVA-16 as a valuable tool for investigations of outbreaks of both human and animal brucellosis. In our study, only 4 of 105 isolates (3.8%) had MLVA-16 genotype 030. It is likely that these cases represented a common-source outbreak or infected the herds of the same genotype. Because consistent epidemiological information for the strains is not routinely available, it is impossible to assess the relationship of the cluster results for these data and outbreaks. An urgent integrated, laboratory-based surveillance is needed to address this important public health gap.

To facilitate outbreak investigation, it has been recommended to use an abbreviated MLVA scheme, omitting testing with panel 1 and 2A since panel 2B is highly polymorphic and potentially more discriminating in determining genetic relationships in regions of endemicity [14]. Some apparently unlinked (epidemiologically or otherwise) isolates had identical MLVA-16 profiles also. This led us to hypothesize that these may represent either epidemiologically unrelated isolates with homoplasy at MLVA-16 loci (most likely panel 2B) or persistent circulating strains causing sporadic infections [3,14]. More detailed genetic investigations such as whole genome sequence comparison, should clarify these relationships.

Results of genotyping confirmed a laboratory-acquired *Brucella *infection. Laboratory workers who handle infected specimens are at high risk of acquiring *Brucella *infection, as suggested by the numerous cases of laboratory-acquired brucellosis reported in the literature [15]. We report a case of brucellosis affecting a hospital microbiology laboratory technician in Beijing, a non-endemic area of China.

Human infection with the vaccine strain M5 in China has not been reported. However, in the previous reports, strains were only biotyped using conventional methods and no direct molecular linkage was shown between the isolated and commercial M5 vaccine strain. In this study, LB 10-01 has the identical genotype with M5. This suggests that LB 10-01 might be that a wild-type biovar 1 evolved with a pattern identical to M5 or that the original strain from which M5 was developed still is transmitted. Results obtained by Garcia-Yoldi et al. confirmed *B. melitensis *vaccine strain Rev 1 group as assayed by MLVA is genetically very homogeneous [16].

## Conclusion

In conclusion, the MLVA assay is very promising to be used as a simple molecular tool for genotypes distribution of *Brucella *isolates from an endemic region, and might be useful for trace back investigations in non-endemic areas.

## Methods

### Bacterial strains and DNA preparation

A total of 104 *B. melitensis *strains used in the study were isolated from clinical samples (102 from blood, and 2 from bone marrow). The samples were collected as part of standard patient care between 1957 and 2010 and were fully de-identified. So any ethical approval was not required for the use of these samples. *B. melitensis *biovar 1 vaccine strain M5 was also included in this study (Table 2). Bacterial isolates were cultured on Trypticase soy agar containing 5% sheep blood (BD Diagnostic Systems, China Ltd., China) at 37°C for 48 h. All isolates were identified as *Brucella *species (biovar) on the basis of classical identification procedures: CO_2 _requirement, H_2_S production, inhibition of growth by basic fuchsin and thionin, agglutination with monospecific antisera and phage typing [17]. Total genomic DNA was extracted with the DNeasy Blood & Tissue Kit (Qiagen China Ltd., China) by following the manufacturer's protocol for extraction of genomic DNA from Gram-negative bacteria. Species-level identification was undertaken by the AMOS-PCR assay [18].

**Table 2:** The 105 *B. melitensis *isolates examined in this study

Geographical origin	Year	No. of isolates	Panel 1 Genotypes*
Inner Mongolia	1955-2006	26	42,63
Qinghai	1965	1	42
Henan	1963,1982	2	42,43
Shanxi	1979-2009	11	42,43,45,63
Shandong	1973,2005	3	42
Shan'xi	1962,2008	5	42
Hebei	2009	1	42
Liaoning	2005	2	42
Guangxi	1961	1	58
Zhejiang	2005,2009	3	42
Fujian	2009	3	42,58
Yunnan	2009	2	58
Beijing	2006	1	42
Guangdong	2006-2010	39	42, 43, 63, CN-1
Hunan	2008	1	42
Jilin	1971	1	42
Tianjin	2010	1	42
Shanghai	2007	1	63
Heilongjiang	1962	1	42

*genotype 42 (1-5-3-13-2-2-3-2), genotype 43 (1-5-3-13-3-2-3-2),

genotype 45 (1-5-3-12-2-2-3-2), genotype 58 (1-5-3-13-3-1-3-2)

genotype 63 (1-5-3-13-2-3-3-2), genotype CN-1 (1-5-3-13-2-1-3-2)

### MLVA-16 genotyping scheme

MLVA was performed as previously described [11]. The sixteen primer pairs were divided into three groups as previously described: panel 1 (8 loci including bruce06, bruce08, bruce11, bruce12, bruce42, bruce43, bruce45, and bruce55), panel 2A (3 loci including bruce18, bruce19, and bruce21), and panel 2B (5 loci including bruce04, bruce07, bruce09, bruce16, and bruce30). PCR conditions were as follows: initial denaturation at 94°C for 3 min, and then 30 cycles of 94°C for 30 s, 60°C for 30 s and 72°C for 50 s. Five microliters of the amplification products were loaded in to 2% (panel 1) and 3% (panels 2A and 2B) agarose gels containing ethidium bromide (0.5 μg/ml), visualized under UV light, and photographed. The reference strain *B. melitensis *16 M, for which the precise molecular mass is known for each primer pair locus, was used for size comparison. To determine the number of repeats from the sample products, PCR products were purified and directly sequenced using an ABI Prism Big Dye Terminator (v3.1) cycle sequencing ready reaction kit (v5.0). The PCR products of samples were sequenced and the sequences were compared to that of *B. melitensis *16 M.

### Analysis of MLVA data

All data were analyzed using BioNumerics version 5.1 software (Applied Maths, Belgium). Clustering analysis was based on the categorical coefficient and unweighted pair group method using arithmetic averages (UPGMA) method. Polymorphism at each loci was quantified using Nei's diversity index, available in the website of HPA http://www.hpa-bioinformatics.org.uk/cgi-bin/DICI/DICI.pl[19]. Resultant genotypes were compared using the web-based Brucella2010 MLVA database http://mlva.u-psud.fr/.

## Authors' contributions

JH did most of the typing work and wrote the report. ZHY, TGZ and PDR prepared the DNA samples. FMG, MJC and YRP were in charge of epidemiological investigation and collection of Inner Mongolia strains. CJD, KCW and DXL were in charge of epidemiological investigation and collection of Guangdong strains. CBY managed the project. All authors read and approved the final manuscript.
